# Chinese Medicines Improve Perimenopausal Symptoms Induced by Surgery, Chemoradiotherapy, or Endocrine Treatment for Breast Cancer

**DOI:** 10.3389/fphar.2019.00174

**Published:** 2019-03-15

**Authors:** Shuo Wang, Hongsheng Lin, Weihong Cong

**Affiliations:** ^1^Laboratory of Cardiovascular Diseases, Xiyuan Hospital, China Academy of Chinese Medical Sciences, Beijing, China; ^2^Department of Oncology of Integrative Chinese and Western Medicine, China-Japan Friendship Hospital, Beijing, China; ^3^Department of Oncology, Guang'anmen Hospital, China Academy of Chinese Medical Sciences, Beijing, China

**Keywords:** Chinese medicine, herbal medicine, complementary therapies, breast cancer, perimenopausal symptoms, quality of life

## Abstract

The application of surgery, chemoradiotherapy, and endocrine treatment successfully increases survival rates of breast cancer patients. However, perimenopausal symptoms, the main side effects of these treatments, often afflict patients and reduce their quality of life. Perimenopausal symptoms include vasomotor symptoms, sleep problems, arthromuscular symptoms, and osteoporosis. Currently, there are no satisfactory treatments for perimenopausal symptoms that result from these treatments. Therefore, alternative and complementary therapies including herbal medicines represented by Chinese medicines (CMs), acupuncture, massage, and psychotherapy are increasingly being expected and explored. In this paper, we review the effects and potentials of several CM formulae, along with some active ingredients or fractions from CMs, Chinese herbal extracts, and other herbal medicines, which have drawn attention for improving perimenopausal symptoms in breast cancer patients. We also elaborate their possible mechanisms. Moreover, further studies for evaluation of standardized clinical efficacy should be scientifically well-designed and continuously performed to investigate the efficacy and mechanisms of CMs for perimenopausal symptoms due to breast cancer therapy. The safety and value of estrogen-containing CMs for breast cancer should also be clarified.

## Introduction

With the comprehensive applications of surgery, radiotherapy, chemotherapy, and endocrine therapy, more women are becoming long-term breast cancer survivors (Bouzbid et al., 2018). However, the increased survival rate is not always accompanied by an improved quality of life. Many breast cancer survivors are plagued by perimenopausal symptoms caused by these treatments, such as vasomotor symptoms, sleep problems, unhealthy emotions, sexual dysfunction, arthromuscular symptoms, and osteoporosis (Hickey et al., 2008; Jeruss and Woodruff, 2009; Park I. H. et al., 2012). After undergoing bilateral oophorectomy, most patients develop severe and sustained hot flushes and other menopausal symptoms (Bachmann, 1999). Chemotherapy and pelvic radiotherapy inhibit ovarian function and cause premature menopause, which adversely affect fertility and sexual function in young breast cancer patients and thus cannot be ignored (Azim et al., 2011). These young survivors may also experience secondary problems in their cardiovascular or skeletal systems (Jeruss and Woodruff, 2009). Although the clinical application of endocrine drugs has greatly improved the survival rates of patients with hormone-dependent breast cancer and reduced the risk of recurrence and metastasis (Fisher et al., 2001), 63.7% of patients taking tamoxifen and 72.7% of patients taking the aromatase inhibitor letrozole have been shown to develop cardiovascular and cerebrovascular events or perimenopausal symptoms including hot flushes, night sweats, arthralgia, and myalgia (Breast International Group (BIG) 1-98 Collaborative Group et al., 2005). As many as one-fifth of breast cancer patients consider stopping endocrine therapy because of their menopausal symptoms (Fellowes et al., 2001), which affects treatment adherence and greatly limits efficacy.

Physical discomfort, bad moods, and social embarrassment due to premature menopause, as well as the long-term, repeated menopausal symptoms in breast cancer patients, cause significant decline in quality of life (Schover, 1994; Gracia and Freeman, 2004). Perimenopausal symptoms are among the most common adverse effects of breast cancer treatment in women of various ages. However, at present, few drugs are available that effectively treat perimenopausal symptoms due to breast cancer therapy. Symptomatic treatment is mainly used in clinical practice, including hormone replacement therapy (HRT), selective serotonin reuptake inhibitors (SSRIs), selective serotonin-norepinephrine reuptake inhibitors (SNRIs), vitamin E, and oryzanol. However, these treatments have many problems in their practical applications. HRT is controversial because it may increase the risk of thromboembolic disease, stroke, breast cancer, endometrial cancer, and ovarian cancer (Rossouw et al., 2002; Archer and Oger, 2012; Henderson and Lobo, 2012; Lee et al., 2016; Sjögren et al., 2016). Specifically, HRT is not recommended for patients with hormone-dependent breast cancer, while recent studies have suggested that its safety should be reconsidered (Fahlén et al., 2013). SSRIs and SNRIs have some effects on perimenopausal symptoms of breast cancer but can cause serious adverse effects such as constipation, dry mouth, and decreased appetite, thus limiting their clinical applications (Stearns and Loprinzi, 2003; Sturdee, 2008; Hall et al., 2011). Other treatments, such as vitamin E and oryzanol, either have poor clinical efficacy or remain in the research phase (Barton et al., 1998).

Therefore, with increasing evidence related to their improvement of perimenopausal symptoms associated with breast cancer therapy, alternative, and complementary therapies, such as herbal medicines including Chinese medicines (CMs), acupuncture, massage, and psychotherapy, are attracting increasing attention (Hachul et al., 2014; Lesi et al., 2016; van Driel et al., 2018). CMs and other herbal medicines in particular, are used worldwide to alleviate menopausal symptoms (Hall et al., 2011; Lin et al., 2017; Moore et al., 2017). However, studies are insufficient regarding the existing perimenopausal symptom-related interventions for those with breast cancer, and the efficacy and safety of these interventions remain to be clarified. In this paper, we review the effects and potentials of several CM formulae, along with some active ingredients or fractions from CMs, Chinese herbal extracts, and other herbal medicines (Table 1) that have gained attention for improving perimenopausal symptoms due to breast cancer therapy in current clinical and experimental studies. We also elaborate their possible mechanisms to provide a reference for future studies and clinical applications.

**Table 1:** CM formulae, active ingredients or fractions from CMs, Chinese herbal extracts, and other herbal medicines for perimenopausal symptoms in breast cancer.

	**Name**
CM formula	Shugan Liangxue Decoction Erxian Decoction Xiaoyao Powder
Active ingredient or fraction from CM	Tenuigenin Resveratrol Genistein
Chinese herbal extract	*Salvia miltiorrhiza* Bge. extract Ginkgo biloba extract
Other herbal medicine	Black cohosh Red clover *Humulus lupulus* L.

## CM Formulae

### Shugan Liangxue Decoction

Shugan Liangxue Decoction is a prescription developed by Professor Pingping Li of the Department of Integrated Chinese and Western Medicine of Beijing Cancer Hospital. It mainly comprises *Bupleurum chinense* DC., *Arnebia euchroma* (Royle) Johnst., *Paeonia lactiflora* Pall., *Paeonia suffruticosa* Andr., *Cynanchum atratum* Bge., and *Schisandra chinensis* (Turcz.) Baill (Table 2).

**Table 2:** Composition of Chinese medicine formulae.

**CM formula**	**Composition of CM formula**
Shugan Liangxue Decoction	*Bupleurum chinense* DC., *Arnebia euchroma* (Royle) Johnst., *Paeonia lactiflora* Pall., *Paeonia suffruticosa* Andr., *Cynanchum atratum* Bge., and *Schisandra chinensis* (Turcz.) Baill.
Erxian Decoction	*Curculigo orchioides* Gaertn, *Epimedium brevicornum* Maxim., *Morinda officinalis* How, *Phellodendron chinense* Schneid., *Anemarrhena asphodeloides* Bge., and *Angelica sinensis* (Oliv.) Diels.
Xiaoyao Powder	*Bupleurum chinense* DC., *Angelica sinensis* (Oliv.) Diels, *Paeonia lactiflora* Pall., *Atractylodes macrocephala* Koidz., *Poria cocos* (Schw.) Wolf, *Glycyrrhiza uralensis* Fisch., *Zingiber officinale* Rosc., and *Mentha haplocalyx* Briq.

Experimental studies have confirmed that Shugan Liangxue Decoction reduced tumor volumes in nude mice with or without ovariectomies. The decoction was also shown to dose-dependently downregulate proliferation of estrogen receptor (ER)-positive breast cancer cells (Fu and Li, 2011; Zhou et al., 2014) and with no significant estrogenic activity (Zhang and Li, 2009; Zhou et al., 2015). Its antitumor activity may be related to its inhibiting key estrogen synthetase, such as aromatase and steroid sulfatase (STS) (Zhang and Li, 2010; Zhou et al., 2014), and may also be related to its selective inhibition of estrogen receptor alpha (ERα) (Zhou et al., 2018). Shugan Liangxue Decoction has no significant influence on the levels of tamoxifen or its metabolites in the human body (Sun and Li, 2009). *In vivo* studies in mice have shown a synergistic effect when Shugan Liangxue Decoction is used with tamoxifen, as it enhances anti-tumor effect of tamoxifen (Wu and Li, 2008) and alleviates tamoxifen's side effects on endometrial thickening (Li et al., 2003). In addition, Shugan Liangxue Decoction combined with anastrozole promotes osteoblast proliferation, enhances osteogenesis (Zhou et al., 2015), and improves bone metabolism (Liu et al., 2009), suggesting that Shugan Liangxue Decoction may improve bone loss caused by endocrine drugs.

Clinical studies have confirmed that Shugan Liangxue Decoction alleviates hot flushes and insomnia in breast cancer patients taking tamoxifen. A randomized, double-blind, placebo-controlled study (Sun et al., 2009) enrolled 73 breast cancer patients (the treatment vs. the control: 37 vs. 36) who developed hot flushes after taking tamoxifen. The patients were continuously treated for 21 days, and the results showed that the proportion of patients in the treatment group whose hot flashes disappeared was 15.2% (vs. 0% in the control group), and the improvement rate was 57.6% (vs. 30.3% in the control group). Further, the proportions of patients with sleep improvement in the treatment and control groups were 63.6 and 39.4%, respectively. All indicators in the treatment group were significantly better than those in the control group. Serum estradiol levels of patients in the treatment group did not significantly change before or after treatment, and no adverse reactions were noted. A similar study (Xue D. et al., 2011) enrolled and analyzed 60 breast cancer patients receiving adjuvant endocrine therapy, of whom 32 patients received Shugan Liangxue Decoction for 6 months per year for over 2 years in addition to the endocrine therapy, while 28 patients received endocrine therapy alone. Such long-term use of Shugan Liangxue Decoction significantly improved patients' hot flushes and sleep without obvious toxicity. Furthermore, the decoction did not affect tumor recurrence or metastasis.

### Erxian Decoction

Erxian Decoction was created by Professor Berna Zhang of the Shuguang Hospital of Shanghai University of Traditional Chinese Medicine. It consists of *Curculigo orchioides* Gaertn, *Epimedium brevicornum* Maxim., *Morinda officinalis* How, *Phellodendron chinense* Schneid., *Anemarrhena asphodeloides* Bge., and *Angelica sinensis* (Oliv.) Diels (Table 2). It is mainly used for menopausal syndrome (Zhong et al., 2013) and is also often used for osteoporosis (Li et al., 2017) and premature ovarian failure (Hu et al., 2013). For decades, Erxian Decoction has been widely used to improve various menopausal symptoms, such as hot flushes, night sweats, insomnia, and depression, due to its definite therapeutic effect with no severe adverse reactions reported (Chen et al., 2008). Recently, network pharmacology studies suggested that about 20 compounds in Erxian Decoction may be the potentially effective ingredients in relieving menopausal symptoms (Wang et al., 2015).

Clinical studies on Erxian Decoction have suggested that it positively affects perimenopausal symptoms in breast cancer patients. One randomized controlled trial (Shao et al., 2015) compared the clinical efficacy of Erxian Decoction combined with tamoxifen vs. tamoxifen alone in treating premenopausal patients with advanced breast cancer (59 cases in each group). The results showed that the total score of CM symptoms, including fatigue, loss of appetite, hot flashes, night sweats, and sleep quality in the Erxian Decoction group were significantly improved after 2 months of treatment. Long-term follow-up also showed that the duration of taking tamoxifen in the Erxian Decoction group was significantly longer than that in the control group. Erxian Decoction can also improve menopausal symptoms, including hot flushes, night sweats, and dysphoria, in breast cancer patients with amenorrhea after postoperative chemotherapy (Liu et al., 2007).

*In vivo* studies showed that Erxian Decoction increased serum estrogen levels by upregulating ovarian aromatase and phosphorylated protein kinase B (p-PKB), thereby alleviating menopausal symptoms (Sze et al., 2009; Wang et al., 2017). *In vitro* studies confirmed that Erxian Decoction could stimulate estrogen production and inhibit proliferation induced by estrogen and metastasis of breast cancer cell as well (Gao et al., 2016; Wang et al., 2017). Erxian Decoction also protected ovaries from chemotherapy injuries (Yuan et al., 2011; Yang et al., 2016) and had less impact on the uterus, mammary gland and vagina of ovariectomized rats (Xue et al., 2012), thus indicating its safety. In addition, Erxian Decoction reduced levels of serum total cholesterol, low-density lipoprotein cholesterol, and modulated blood lipid levels in postmenopausal rats (Sze et al., 2011). Erxian Decoction could also improve osteoporosis in ovariectomized rats (Xue L. et al., 2011) and regulate osteoblast activity and bone metabolism (Zhu et al., 2010), and its bone protection may be related to the ER-mediated signaling pathway (Wong et al., 2014).

### Xiaoyao Powder

Xiaoyao Powder is derived from the *Prescriptions of the Bureau of Taiping People's Welfare Pharmacy* issued by the government in 1151. It consists of *Bupleurum chinense* DC., A*ngelica sinensis* (Oliv.) Diels, *Paeonia lactiflora* Pall., *Atractylodes macrocephala* Koidz., *Poria cocos* (Schw.) Wolf, *Glycyrrhiza uralensis* Fisch., *Zingiber officinale* Rosc., and *Mentha haplocalyx* Briq (Table 2). Depending on the patient's symptoms, in clinical practice, corresponding CMs are added to the Xiaoyao Powder under the guidance of the CM principle for syndrome differentiation and treatment to create the modified Xiaoyao Powder. Xiaoyao Powder and modified Xiaoyao Powder are mainly used to treat menopausal syndrome and premenstrual syndrome (Scheid et al., 2010; Chen H. Y. et al., 2015) and are frequently used for cancer, insomnia, functional dyspepsia, and poststroke depression (Qin et al., 2009; Bai et al., 2010; Lee K. H. et al., 2013; Tsai et al., 2014; Liao et al., 2016). Clinical applications confirmed that Xiaoyao Powder and modified Xiaoyao Powder effectively improved perimenopausal symptoms, such as insomnia and emotional disorder (Chen et al., 2011; Terauchi et al., 2011; Wang et al., 2014).

A 10-year analysis of 20,466 breast cancer patients treated with tamoxifen showed that more than half the subjects had ever used CMs, in which modified Xiaoyao Powder had the highest utilization rate, nearly one-third. The analysis showed that application of CMs reduced the risk of endometrial cancer induced by tamoxifen (Tsai et al., 2014). What is the effect of modified Xiaoyao Powder on perimenopausal symptoms in breast cancer patients? A randomized controlled trial (Sun and Zhang, 2013) observed modified Xiaoyao Powder's therapeutic effects on breast cancer patients receiving tamoxifen and enrolled 31 patients administered with the modified Xiaoyao Powder and 30 cases with tamoxifen alone. After 2 months of treatment, the Kupperman Index of the modified Xiaoyao Powder group was significantly lower than that of the control group. Meanwhile modified Xiaoyao Powder did not affect the estrogen levels. Other clinical studies (Xu et al., 2005; Zhang and Zheng, 2012; Fu, 2016; Zhao et al., 2017) also showed that modified Xiaoyao Powder produced some improvement of perimenopausal symptoms of breast cancer and had good safety; however, more high-quality studies are needed to support the present research conclusion.

*In vivo* and *in vitro* studies have shown that Xiaoyao Powder induced apoptosis and autophagy of hormone-dependent breast cancer MCF-7 cells (Wang et al., 2009; Li et al., 2016a,b) and inhibited hormone-dependent and hormone-independent growth of breast tumor (Chen et al., 2012; Qi et al., 2015; Li et al., 2016a,b). Studies evaluated estrogen-like effects of flavonoid components in Xiaoyao Powder and found that they enhanced ERα and ERβ expression and promoted MCF-7 cell proliferation (Chen et al., 2016). While some researchers (Song and Li, 2013) confirmed that although *Angelica sinensis* (Oliv.) Diels in Xiaoyao Powder revealed phytoestrogen-like effects, Xiaoyao Powder itself had no effect on tumor growth and did not exhibit estrogen-like effects. Such inconformity also reflects the complexity of the efficacy of CM formulae containing complex mixtures of naturally-occurring chemicals, which needs further exploration and more evidence to confirm the effects of Xiaoyao Powder on estrogen levels and ERs.

## Active Ingredients Or Fractions From CMs

### Tenuigenin

Tenuigenin is one of the main active ingredients of one CM herb, *Polygala tenuifolia* Wild (Table 3). Pharmacological studies have shown that tenuigenin plays roles in neuroprotection, memory and cognitive improvement, and antioxidation (Sun et al., 2007; Chen et al., 2010; Liang et al., 2011; Huang et al., 2013). Clinically, the reduction of estrogen, such as after ovariectomy, may lead to cognitive impairment (Walf et al., 2009; Su et al., 2010), and breast cancer patients after ovariectomy may have problems with learning and memory. Studies (Cai et al., 2013) have shown that tenuigenin improved memory and cognitive deficits in ovariectomized mice, which may be related to its reducing the loss of nitric oxide synthase (NOS)-positive neurons and improving the changes in synaptic morphology of the hippocampal CA1 area induced by ovariectomy. This suggests that the therapeutic effects of tenuigenin on menopausal neurological symptoms and cognitive dysfunction after ovariectomy are worth further study.

**Table 3:** Information of active ingredients of Chinese herbal medicine.

**Active ingredient of Chinese herbal medicine**	CAS Rn^*^	**Molecular structure of active ingredient**
Tenuigenin(Senegenin)	667438-01-9 (2469-34-3)	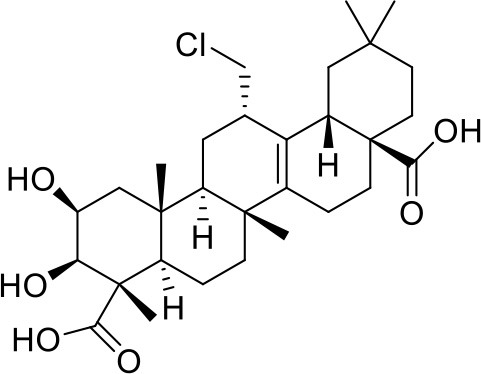
Resveratrol	501-36-0	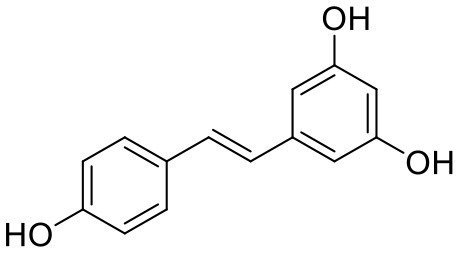
Genistein	446-72-0	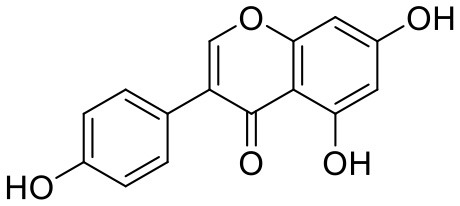

* *CAS Rn: Chemical Abstracts Service Registry Number*.

### Resveratrol

Resveratrol is widely present in common plants, such as grapes, peanuts, and *Polygonum cuspidatum* Sieb. et Zucc. (Li T. K. et al., 2016), and has several biological activities including antitumor and anti-cardiovascular disease action, neuroprotection, antioxidation, and liver protection (Liman et al., 2000; Athar et al., 2007; Rivera et al., 2008; Liu et al., 2011) (Table 3).

Many studies have been conducted on resveratrol improving menopausal symptoms. A 14-week randomized double-blind placebo-controlled study enrolled 80 postmenopausal women receiving trans-resveratrol (75 mg, twice daily) and suggested that resveratrol relieved the chronic joint pain in menopausal women (Rhx et al., 2017). Other randomized controlled trials found that resveratrol could improve cerebrovascular and cognitive function (Evans et al., 2017), decrease the number of vasomotor symptoms, and alleviate the degree of hot flashes in menopausal women (Leo et al., 2015).

Animal experiments verified that oral intake of resveratrol had less effect on the endometrium (Zhang W. Z. et al., 2008). *In vivo* and *in vitro* studies have also demonstrated that resveratrol has anti-breast cancer effects (Scarlatti et al., 2008; He et al., 2011; Fu et al., 2014). Although resveratrol is expected to improve menopausal symptoms in breast cancer patients, it remains highly controversial because of its possible estrogen-like effect at present. Resveratrol can competitively bind to ERs, and researchers suggest that such estrogen-like effects may be related to its ovarian protection (Banu et al., 2016) and improvement of menopausal symptoms. Studies in the 1990s have found that resveratrol had the anti-estrogen effect and dose-dependent inhibition of the growth of ER-positive breast cancer MCF-7 cells (Lu and Serrero, 1999). However, in additional studies, researchers discovered resveratrol's dual identity as both estrogen agonist and antagonist, which might account for the conflicting results in studies of resveratrol and estrogen-related cancers. Therefore, determining resveratrol's safety in different breast cancer patient subgroups will be the primary task of future clinical research (Bartolacci et al., 2018).

### Genistein

The adverse effects of HRT are mainly related to the activation of estrogen receptor subtype ERα, which has bottlenecked HRT use for menopausal diseases and makes phytoestrogens attract more attention than ever. Soy isoflavones are well-recognized and extensively studied phytoestrogens (Setchell, 1998). In Asian countries, each woman consumes ~50 mg of isoflavones per day, much higher than that in western countries (Messina et al., 2006). This soy-rich diet for Asian women is considered to play an important role in reducing breast cancer incidence (Tham et al., 1998). Genistein is a major natural soy isoflavone (Sarkar and Li, 2002) (Table 3). Many studies have shown that genistein plays anti-breast cancer roles by regulating cell cycles, inhibiting cell proliferation (Pagliacci et al., 1994; Upadhyay et al., 2001), inducing apoptosis (Li et al., 1999b), and inhibiting tumor angiogenesis and metastasis (Li et al., 1999a).

Genistein is also closely associated with the improvement of perimenopausal symptoms. A 12-week, multicenter, randomized, placebo-controlled clinical study examined the effect of genistein on improving symptoms in postmenopausal women. Eighty-four postmenopausal women received placebo treatment (42 cases) or a single 3,002 mg dose of synthetic genistein (40 cases). The results showed that the number and duration of hot flashes in the genistein group were significantly decreased, and no statistical differences were found in 17β-estradiol, follicle stimulating hormone (FSH), endometrial thickness, or adverse events compared with the placebo group (Evans et al., 2011). Genistein also improves postmenopausal osteoporosis, vaginal atrophy-related symptoms, dry eye syndrome, and cardiovascular risk (Crisafulli et al., 2005; Le et al., 2011; Shao et al., 2012; Arcoraci et al., 2017). Despite all this, limited by its identity as a phytoestrogen, genistein's risk and safety for clinical use in the treatment of breast cancer patients are still yet to be fully considered. Genistein not only antagonizes ERα and its mediated signaling pathway (Choi et al., 2014) but also has a stronger affinity for estrogen receptor beta (ERβ) than for ERα (Chang et al., 2008), which is a natural selective estrogen receptor modulator (SERM) (Sareddy and Vadlamudi, 2015). However, some experimental studies (Pons et al., 2016) suggest that the ERα/ERβ ratio should be considered carefully when genistein is used to treat breast cancer patients. Using genistein in patients with a high ERα/ERβ ratio may be counterproductive. Thus, more experimental and clinical studies are needed to verify the effect and safety of genistein for perimenopausal symptoms of breast cancer.

## Chinese Herbal Extracts

### *Salvia miltiorrhiza* Bge. Extract

**Figure 1:**
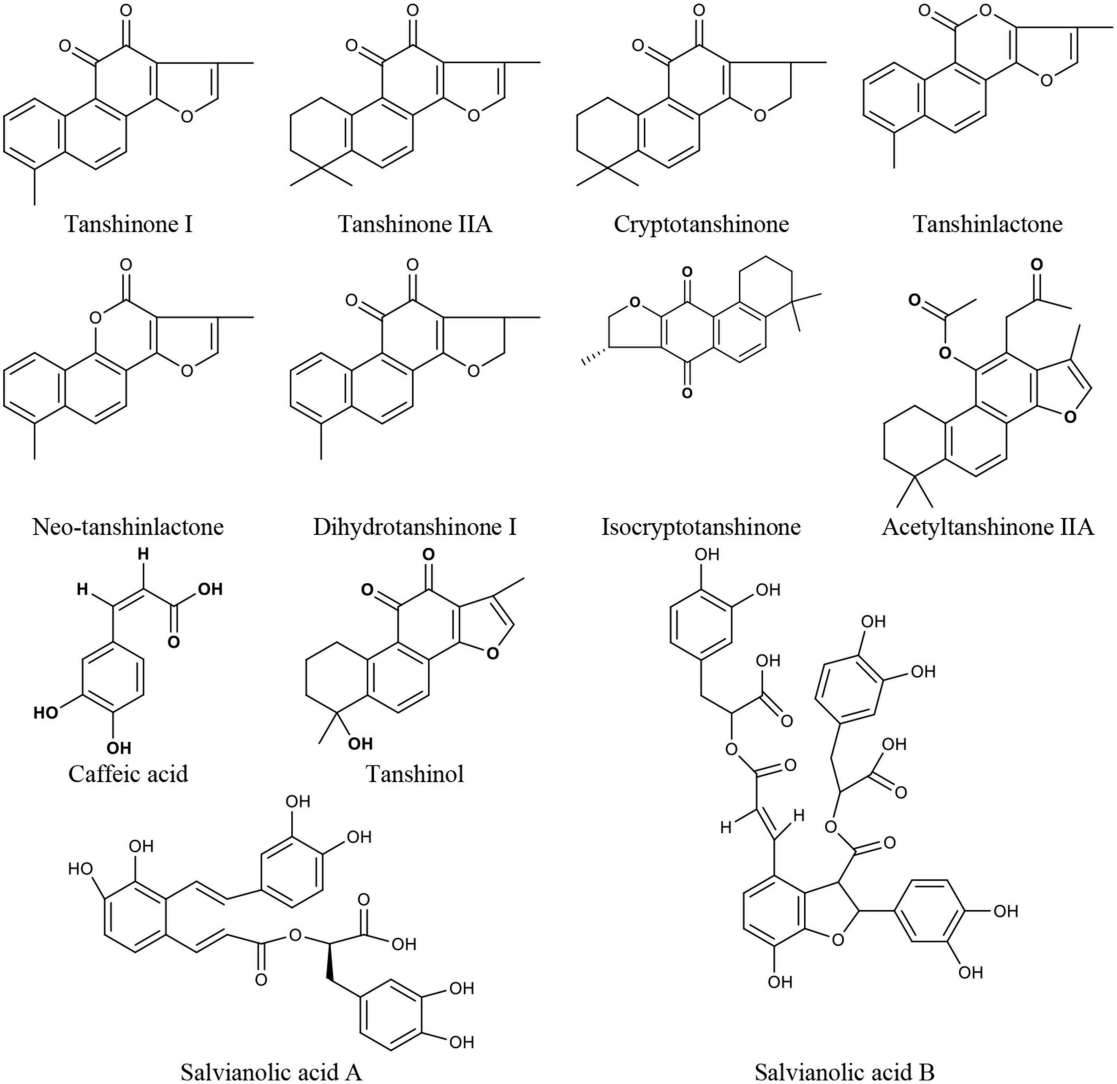
The chemical structures of compounds isolated from *Salvia miltiorrhiza* Bunge.

*Salvia miltiorrhiza* Bge. is a CM herb with the function of promoting blood circulation to remove blood stasis, which has a long history of clinical use, mainly for cardiovascular and cerebrovascular diseases but also in liver and kidney diseases (Wang, 2010; Sun et al., 2015). In China, it is also frequently used for menopausal disorders (Guo et al., 2014; Kwok et al., 2014). There are *Salvia miltiorrhiza* Bge. aqueous extracts (mainly containing tanshinol, caffeic acid, salvianolic acid A, and salvianolic acid B) and alcoholic extracts (mainly containing tanshinone I, tanshinone IIA, cryptotanshinone, tanshinlactone, acetyltanshinone IIA, isocryptotanshinone, dihydrotanshinone I, and neo-tanshinlactone) (Figure 1). Modern research demonstrates that many compounds of *Salvia miltiorrhiza* Bge. extract display anti-tumor activity (Zhang et al., 2012; Chen et al., 2014; Sung et al., 2015; Shen et al., 2016), which gives promising prospects for the treatment of breast cancer (Yang et al., 2010; Gong et al., 2012; Kim et al., 2017). Furthermore, *Salvia miltiorrhiza* Bge. extract has also been shown to prevent bone loss, reduce serum triglyceride and low-density lipoprotein cholesterol levels (Zhang et al., 2016), and protect vascular function (Li et al., 2013) in ovariectomized rats. Some researchers suggest that *Salvia miltiorrhiza* Bge. might be a potential SERM with a strong affinity for ERβ (Zhang et al., 2016), and its heart and bone protective effects are mediated by ERs (Weng et al., 2013; Xu et al., 2017).

As the representative active ingredient of the *Salvia miltiorrhiza* Bge. extracts, Tanshinone IIA is chosen as the biomarker for quality control of Danshen in the 2010 edition of Chinese Pharmacopeia. Tanshinone IIA exhibited anti-estrogen properties and inhibited the growth of breast cancer cells (Zhao et al., 2015). Many effects of tanshinone IIA, including cardiovascular protection (Xu et al., 2009; Fan et al., 2011), neuroprotection (Shen et al., 2011), and antiosteoporosis (Kwak et al., 2006), are closely related to menopausal problems. Tanshinone I could significantly induce the apoptosis of ER-positive (MCF-7) and ER-negative (MDA-MB-231) cells (Nizamutdinova et al., 2008), and inhibit the growth of breast cancer cells by the downregulation of Aurora A (Gong et al., 2012). It also has neuroprotective effects (Lee J. C. et al., 2013; Jing et al., 2016). Cryptotanshinone could lead to the apoptosis of MCF-7 cells as a potent stimulator of ER stress mediated by mitogen-activated protein kinases (Park I. J. et al., 2012), and inhibit the growth of ERα-positive breast cancer cells by competitively binding to ERα to suppress ER transcriptional activity (Li et al., 2015). It also possesses cardiovascular protection and neuroprotective effects (Yoo and Park, 2012; Oche et al., 2016). Neo-tanshinlactone inhibited growth and induced apoptosis of ER-positive breast cancer cells through decreasing ERα expression levels and transcriptional activities (Lin et al., 2016). Thus, *Salvia miltiorrhiza* Bge. and its active ingredients might be the potential drugs for treating perimenopausal symptoms due to breast cancer therapy.

### Ginkgo Biloba Extract

**Figure 2:**
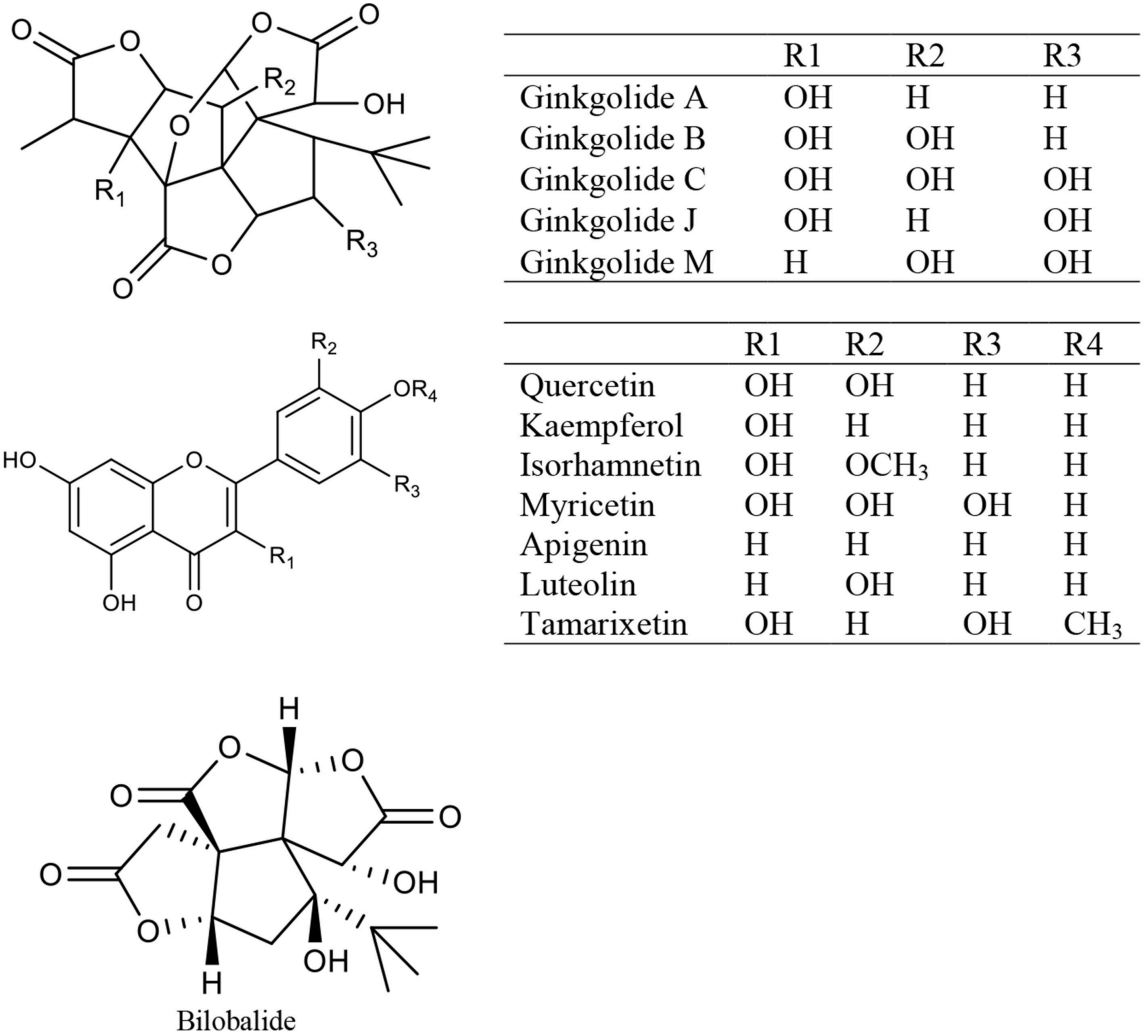
The chemical structures of compounds isolated from Ginkgo biloba extract.

Ginkgo biloba extract (GBE) is a mixture of medicinal ingredients extracted from dried *Ginkgo biloba* L leaves. The predominant pharmacologically active constituents of GBE were identified to be flavonols (quercetin, kaempferol, isorhamnetin, myricetin, apigenin, luteolin, and tamarixetin) and terpene trilactones (ginkgolide A, ginkgolide B, ginkgolide C, ginkgolide J, ginkgolide M, and bilobalide) (Mohanta et al., 2014) (Figure 2). GBE has many effects, such as antioxidation, antiplatelet aggregation, anti-inflammation, and antitumor activity (Packer, 1994; Duttaroy et al., 1999; Ilieva et al., 2004; Dias et al., 2008; Zhang Y. et al., 2008). Clinically, GBE improves menopausal cognitive function (Yuan et al., 2017). A triple-blind, placebo-controlled trial enrolled 80 healthy female volunteers in which 40 individuals received a dose of 120–240 mg GBE while the other 40 received the placebo daily for 30 days. The results showed that GBE positively affected sexual desire in menopausal women (Pebdani et al., 2014). Experimental studies revealed that GBE reduced body weight and adiposity in ovariectomized rats by downregulating 5-HT levels in the hypothalamus (Banin et al., 2017), inhibited central neurodegeneration (Shi et al., 2010), significantly increased cognitive function in rats (El Tabaa et al., 2017), and controlled bone loss caused by the lack of estrogen (Trivedi et al., 2009). Ginkgo biloba contains phytoestrogens. Researchers (Oh and Chung, 2004) studied the estrogen activity of ginkgo biloba and its main components (kaempferol, quercetin, and isorhamnetin), and found that these components affected both ERα and ERβ but showed greater affinity for ERβ than ERα, and induced the transcription of pS2 gene and progesterone receptor in MCF-7 cells. Recent studies have shown that GBE restrains estrogen-sensitive breast cancer by inhibiting aromatase and estrogen production (Park et al., 2015, 2016), restrains the proliferation of ER-negative breast cancer cells independently of the ERs (Park et al., 2013; Zhao et al., 2013). Additionally, GBE has a synergistic effect with tamoxifen (Dias et al., 2013).

## Other Herbal Medicines

There are also a number of other herbal medicines that have attracted widespread attention for their role in improving perimenopausal symptoms in breast cancer patients.

Black cohosh, a plant that contains many active ingredients such as triterpene glycosides, is one of the most widely used herbs in Europe for relieving menopausal symptoms in women (Reed et al., 2005; Pockaj et al., 2006; Bai et al., 2007). Studies have shown that black cohosh extract improves hot flashes, night sweats, insomnia, anxiety, and other perimenopausal symptoms in breast cancer patients (Vermes et al., 2005; Rostock et al., 2011). It does not increase breast density, endometrial thickness (Hirschberg et al., 2007), or the risk of breast cancer recurrence and metastasis (Henneickevon Zepelin et al., 2007; Obi et al., 2009). Both *in vivo* and *in vitro* studies have indicated that black cohosh extract has no estrogenic activity (And and Henion, 2001; Lupu et al., 2003), while it has demonstrated antiestrogen effects and an inhibiting effect on breast cancer cell proliferation (Bodinet and Freudenstein, 2004; Einbond et al., 2004). There are many theories about its mechanism of action to date. Some scholars believe that black cohosh does not work through ERs but through neurotransmitters such as 5-HT instead (Burdette et al., 2003). It is also hypothesized that the mechanism of action of black cohosh is similar to SERMs according to its clinical estrogen-like effects. However, results of clinical studies on improving menopausal symptoms with black cohosh are sometimes inconsistent, and some studies found its efficacy was not significantly different from placebo treatment (Geller et al., 2009; Fritz et al., 2014; Tanmahasamut et al., 2015). Considering the differences in black cohosh plant types, extracting methods, dosages, and enrolled populations in different studies, its effectiveness is yet to be explored in larger sample clinical trials. With regard to the adverse reactions of black cohosh, in addition to hepatotoxicity (Mahady et al., 2008), a study found that black cohosh increased the incidence of lung metastasis in c-erbB2-positive transgenic breast cancer mice. It suggests that the safety of long-term use of black cohosh products may need further consideration (Davis et al., 2008).

In addition, red clover (*Trifolium pratense* L.), a perennial plant, is employed in improving menopausal symptoms. Some studies showed that red clover isoflavone extract might improve hot flash frequency (van de Weijer and Barentsen, 2002), while a meta-analysis involving 6 randomized studies did not support the conclusion of red clover reducing vasomotor symptoms (Nelson et al., 2006). As for the safety of red clover for breast cancer, several clinical trials reported that red clover showed no significant effect on estradiol increase or on breast and endometrial thickness in postmenopausal women (Charlotte et al., 2004; Powles et al., 2008). Hops (*Humulus lupulus* L.) are an important ingredient in beer brewing and are used in dietary supplements to improve menopausal symptoms in Europe. Currently, there is still not enough clinical evidence to support the beneficial effect of hops on menopausal syndrome (Palmieri et al., 2009; Erkkola et al., 2010). Meanwhile, studies revealed that both red clover extracts and hops extract showed significant ER competitive binding and estrogen-induced gene activation. The red clover extract had nearly a 9-fold preference for ERα compared with ERβ, while the hops extract preferentially bound to ERβ receptor twice as much than to ERα. Both red clover extracts and hops extracts showed equivalent ERα activities (Overk et al., 2005). To get further convictive conclusion, more work should be done on the efficacy and safety of these herbs for perimenopausal symptoms of breast cancer.

## Discussion

How to improve quality of life is a major challenge in treating long-term breast cancer survivors. Perimenopausal symptoms due to breast cancer therapy are the most common problems that plague patients' daily lives. Whether it is premature menopause in young breast cancer patients (Murthy and Chamberlain, 2012), or the exacerbation of menopausal symptoms in perimenopausal breast cancer patients, physical, psychological, and social problems are commonly and severely affect these patients' quality of life (Harris et al., 2002; Crandall et al., 2004; Gupta et al., 2006).

For breast cancer patients, methods for improving perimenopausal symptoms are limited. Thus, most breast cancer survivors would like to seek help from complementary and alternative. But the relevant information on these therapies is sometimes unreliable or contradictory, and even clinicians often cannot provide clear recommendations (Légaré et al., 2007; Suter et al., 2007). Among these therapies, herbal medicines, especially CMs, are natural and have become the major choice for patients (Moore et al., 2017). In particular, CM is good at taking measures according to the variability of an individual. CMs have a long application history of improving menopausal symptoms in Asian countries, including China, and are receiving more attention worldwide.

However, CMs used to improve natural menopausal symptoms are not always suitable for treating perimenopausal symptoms of breast cancer patients. On one hand, HRT use is controversial for natural menopausal women, and some herbal medicines with estrogen-like effects are also questioned (Lin et al., 2017). CM safety must be the primary concern of researchers for hormone-dependent breast cancer. On the other hand, perimenopausal symptoms of breast cancer patients occur after surgery, radiotherapy, chemotherapy, and endocrine therapy and are closely related to impaired ovarian function and the sudden decline of estrogen levels, but other non-estrogen causes are also considered. Whether or not the mechanism of perimenopausal symptoms due to breast cancer therapy differs from that of natural menopause requires further study. Furthermore, for perimenopausal or postmenopausal women, after the diagnosis and treatment of breast cancer, the internal mechanisms of the occurrence and exacerbation of their menopausal symptoms are even more complicated. The complexity and diversity of perimenopausal symptoms make it difficult for single-target drugs to solve all these problems. Thus, the multi-targeted and comprehensive effects of CM formulae present advantages. In clinical practice, especially in China, CM formulae have been widely used to improve perimenopausal symptoms in breast cancer patients, and some positive conclusions have been drawn from existing studies. However, the existing clinical research has many problems that may affect the credibility of conclusions. For example, many studies are of limited quantity and quality, and multicenter and large-scale clinical trials are insufficient. Most studies evaluate the curative effect by the total symptom score, while there is a lack of standardized measurements for a single major symptom. Researchers pay more attention to symptoms such as hot flashes, night sweats, and insomnia rather than emotion and sexual function. The research quality heterogeneity, including the different preparation methods of CM formulae, affects the results' comparability and reliability. The time of research observation and follow-up is often too short and there are few long-term safety indicators related to breast cancer recurrence and metastasis. Furthermore, except for the antitumor effects of CM formulae, there is not enough experimental research on aspects such as its estrogenic or anti-estrogenic activity, impact on the ERs statuses, mammary glands and uterine tissue and interactions with other drugs. CMs are considered to be multi-targeted possibly due to the variety of ingredients that they contain, which is one of the most significant characteristics of CMs. Although not all targets of each CM are identified, many targets of CMs, especially some commonly used ones, have been studied and identified. Due to the growing needs from patients suffering perimenopausal symptoms of breast cancer, further work should be done to find the molecular mechanisms of these formulae for their better use in clinical practice. In addition, there are few classic traditional formulae for perimenopausal symptoms of breast cancer studied so far. The classic formulae are the treasure of traditional Chinese medicine, some of which have long been used for treating female menopausal symptoms. Such classic formulae might be potential research topics of improving menopausal symptoms of breast cancer, which will certainly attract more attention in future. Therefore, extensive applications of CM formulae in perimenopausal symptoms in breast cancer patients still need more supportive data from higher quality, transparent, and in-depth studies.

**Figure 3:**
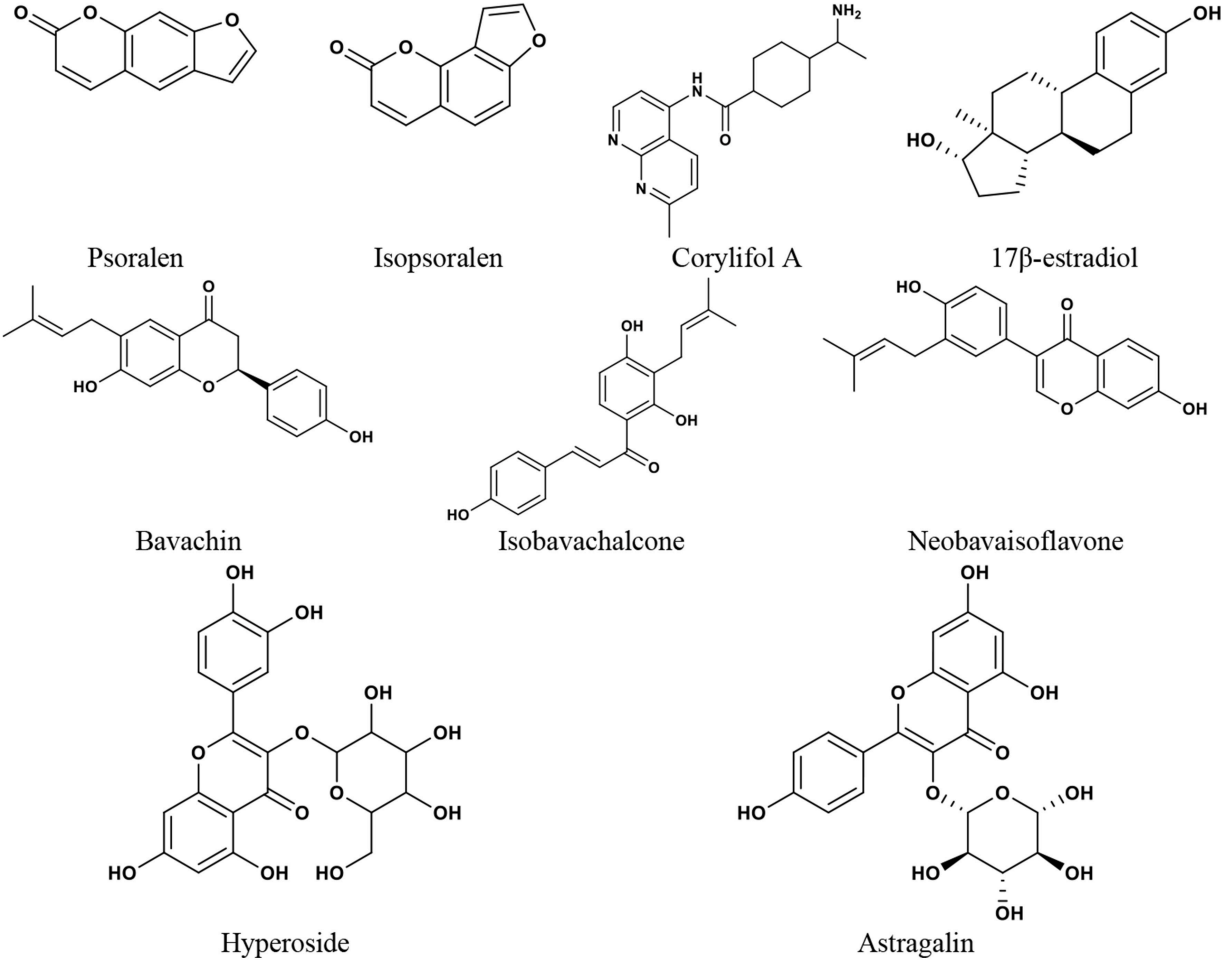
The chemical structures of phytoestrogens and 17β-estradiol reported in the discussion section.

Many CMs commonly used for menopause contain phytoestrogens. Phytoestrogens are similar to endogenous estradiol in structure and can bind to ER to exert estrogenic or anti-estrogenic effects. The major groups of phytoestrogens include isoflavones, coumarins, lignans, and stilbenes (Basu and Maier, 2018), which show different active effects. Both *Psoralea corylifolia* L. and *Cuscuta chinensis* Lam. are Chinese medicines and are often used in the treatment of osteoporosis (Donnapee et al., 2014; Zhang et al., 2016). It was reported that (Xin et al., 2010) the two coumarins in the EtOH extract of *Psoralea corylifolia* L., isopsoralen and psoralen, were selective activators of ERα, which could significantly promote the proliferation of MCF-7 cells. The four flavonoids, isobavachalcone, bavachin, corylifol A, and neobavaisoflavone, could simultaneously activate both ERα and ERβ. All these compounds could exert estrogenic activities through ER, but they may have different biological effects. Yang et al. studied the antiosteoporosis activity of flavonoids in the crude ethanolic extract of *Cuscuta chinensis* Lam. (Yang et al., 2011). It was revealed that kaempferol and hyperoside significantly increased ALP activity in UMR-106 cells and astragalin promoted the proliferation of UMR-106 cells, which showed estrogenic activity. Quercetin and kaempferol showed potent ER antagonist activity by activating ERα/β-mediated AP-1 reporter expression. It was further suggested that the antiosteoporosis effect of *Cuscuta chinensis* Lam. might be closely related to the estrogenic or anti-estrogenic activities of flavonoids (Figure 3). Therefore, phytoestrogens are an important issue when it comes to treating perimenopausal symptoms of breast cancer with CM and other herbal medicines. At present, there are more studies conducted on treating menopausal symptoms with herbs and foods containing phytoestrogens, and many studies have obtained positive results (Bedell et al., 2014; Chen M. N. et al., 2015). However, since phytoestrogens may have similar effects to human endogenous estrogen, as well as the limitation of ethics, data from relevant clinical research on perimenopausal symptoms in breast cancer patients are inadequate. Despite the basic structure of phytoestrogens being similar to that of estradiol, which indicates their estrogen-like properties, phytoestrogen differs from estradiol. Human endogenous estrogen acts mainly via ERα- and ERβ-mediated transcriptional activation or effects (Hillisch et al., 2004). While protecting the cardiovascular, cerebrovascular, nervous, and skeletal systems, human endogenous estrogen has a carcinogenic risk to breasts and the uterus. ERα and ERβ are distributed differently in different tissues. The mammary glands mainly contain ERα, and ERα overactivation is an important factor in the occurrence and development of hormone-dependent breast cancer. As a tissue-specific tumor inhibitor, ERβ has an antiproliferative effect (Nilsson and Gustafsson, 2011). ERβ opposes the effect of ERα by modulating the expression of ERα-regulated genes (Clarke, 2003). Different phytoestrogens have different effects on the two ER subtypes. Studies (Sareddy and Vadlamudi, 2015) have shown that some phytoestrogens exhibit the characteristics of SERMs, which can antagonize ERα or have a higher affinity for ERβ, and can selectively activate the ERβ transcriptional pathway. It may avoid the drawbacks of endogenous estrogen. Genistein interacts with both ERα and ERβ, and has a higher affinity for ERβ (Chang et al., 2008). The affinity of genistein for ERα was 4%, while it was 87% for ERβ, compared with estradiol (Kuiper et al., 1998). Genistein was reported to recruit the steroid receptor coactivator 3 (SRC3) much more efficiently to ERβ than to ERα (Jiang et al., 2013). Meanwhile, genistein could inhibit the expression of ERα, antagonize the signal pathway of ERα, and affect the proliferation and apoptosis of breast cancer cells (Choi et al., 2014). It was also reported that genistein significantly reduced the expression of ERα mRNA and increased the ERβ level in three different ER-positive breast cancer cells, MCF-7, T47D, and 21 PT (Marik et al., 2011). Due to the similarity in structure to the synthetic estrogen diethylstilbestrol, resveratrol interacts with ER and has been designated as the “phytoestrogen” (Mueller et al., 2004). However, resveratrol may be a combination of the agonist and antagonist to estrogen, depending on the dosage and concentration of resveratrol and 17β-estradiol (E2), as well as the expression of ERα and ERβ in tissue cells (Bhat et al., 2001). *Salvia miltiorrhiza* Bge. extract could interact with ERα and ERβ, and significantly induce the expression of ERα/β-estrogen response element (ERE) luciferase reporter gene without side effects on reproductive tissues (Xu et al., 2017). There are many flavonoids in ginkgo biloba extract, which have been demonstrated to affect ERα and ERβ and show a higher affinity for ERβ than for ERα. It can also induce progesterone receptor transcription in MCF-7 cells (Oh and Chung, 2004). In addition to the regulatory effect on estrogen receptor expression, phytoestrogens also inhibit estrogen biosynthesizing enzymes. Promoters I.3 and II are the major promoters directing aromatase expression in breast cancer, and genistein may inhibit the activities of promoters I.3 and II for CYP19 regulation (Chen et al., 1999). A study showed that a methoxy derivative of resveratrol, 3MS, could efficiently inhibit the expression of aromatase protein encoded by CYP19 in MDA-MB- 231 cells (Licznerska et al., 2017). The standard GBE (EGb 761) significantly inhibited aromatase activity and reduced the expression of CYP19 mRNA and CYP19 promoter I.3 and PII (Park et al., 2016). Furthermore, phytoestrogens may inhibit estrogen metabolic enzymes. Genistein impacts the formation of estrogen metabolites and is a potent inhibitor of E1 and E2 sulfation (Poschner et al., 2017). Cytochrome P450 CYP1 family enzymes such as CYP1A1, CYP1A2, and CYP1B1 are important enzymes in estrogen metabolism. It was reported that resveratrol could inhibit dioxin-induced CYP1A1 and CYP1B1 expression levels and recruitment of AHR and ERα in T-47D cells (Macpherson and Matthews, 2010). Meanwhile, many studies have shown that phytoestrogen intake reduces the risk of breast cancer, which may be related to its effects of decreasing estrogen and progesterone levels and reducing endogenous hormonal stimulation in breast tissue (Kumar et al., 2002; Peeters et al., 2004; Touillaud et al., 2005). *In vivo* and *in vitro* studies have also found that some phytoestrogens inhibit breast cancer growth (Li L. et al., 2016). Thus, phytoestrogens with such characteristics would be of great value in treating perimenopausal symptoms due to breast cancer therapy.

Therefore, in this paper, we reviewed the potential roles of CMs in treating perimenopausal symptoms in breast cancer patients, focusing on several CM formulae, along with some active ingredients or fractions from CMs, Chinese herbal extracts and other herbal medicines. We also elaborate their interactions with ERs and anti-breast cancer properties. CM is a kind of medicine originating from the experience of application in humans and has benefitted mankind throughout history. It should be noted that more work on the molecular mechanisms and in-depth pre-clinical studies of CMs are necessary, which will be helpful to explain the biological activity and mechanism, confirm the safety and effectiveness, and determine the ideal dosages for clinical trials and finally for the better use in patients. It is expected that future research will thoroughly investigate the efficacy, effective dose, and adverse reactions of these drugs and their interactions with chemotherapeutics or endocrine drugs, clarify the safety and value of estrogen-containing CMs for breast cancer patients, and screen out drugs with high safety and efficacy in treating perimenopausal symptoms of breast cancer for better clinical use. CMs have been used in clinical practice for a long time and are a potential medicinal source for treating complex diseases. In-depth exploration of the roles, mechanisms and material bases of CMs for perimenopausal symptoms after surgery, radiotherapy, chemotherapy, and endocrine therapy for breast cancer, may provide more choices for patients.

## Author Contributions

SW and WC wrote the manuscript. HL and WC designed and edited the manuscript. All authors contributed to and approved the final version of the manuscript.

### Conflict of Interest Statement

The authors declare that the research was conducted in the absence of any commercial or financial relationships that could be construed as a potential conflict of interest.
